# Glucocorticoids Target Ependymal Glia and Inhibit Repair of the Injured Spinal Cord

**DOI:** 10.3389/fcell.2019.00056

**Published:** 2019-04-24

**Authors:** Craig M. Nelson, Vanda A. Lennon, Han Lee, Randall G. Krug, Aichurok Kamalova, Nicolas N. Madigan, Karl J. Clark, Anthony J. Windebank, John R. Henley

**Affiliations:** ^1^Department of Neurologic Surgery, Mayo Clinic, Rochester, MN, United States; ^2^Department of Laboratory Medicine and Pathology, Mayo Clinic, Rochester, MN, United States; ^3^Department of Neurology, Mayo Clinic, Rochester, MN, United States; ^4^Department of Immunology, Mayo Clinic, Rochester, MN, United States; ^5^Department of Biochemistry and Molecular Biology, Mayo Clinic, Rochester, MN, United States; ^6^Department of Physiology and Biomedical Engineering, Mayo Graduate School, Mayo Clinic College of Medicine and Science, Rochester, MN, United States

**Keywords:** neural regeneration, spinal cord injury, glucocorticoid signaling, Nr3c1, ependymal glia

## Abstract

Following injury, the mammalian spinal cord forms a glial scar and fails to regenerate. In contrast, vertebrate fish spinal cord tissue regenerates significantly to restore function. Cord transection in zebrafish (*Danio rerio*) initially causes paralysis and neural cell death. Subsequently, ependymal glia proliferate, bipolar glia extend across the lesion, and new neurons are born; axons from spared and nascent neurons extend along *trans*-lesional glial bridges to restore functional connectivity. Here we report that glucocorticoids, used in the clinical management of spinal cord injury, directly inhibit neural repair by targeting ependymal glia independently of hematogenous cells and microglia. After transecting injury, the glucocorticoid receptor in ependymal glia is regulated differentially in zebrafish (becoming inactive) vs. the rat (becoming active). Glucocorticoid blockade of neural regeneration via a direct effect on ependymal glia has important therapeutic implications for the putative benefit of corticosteroids in early management of spinal cord injury.

## Introduction

Spinal cord injury causes life-long physical disability. The goal of contemporary treatment is to limit morbidity by stabilizing the injury and managing inflammation; functional restoration is unattainable (Bracken et al., 1990, 1997; Eck et al., 2006). For many decades, glucocorticoid (GC) therapy has been considered a means for limiting tissue damage and loss of function after spinal cord injury (Bracken et al., 1984, 1990, 1997; Hurlbert, 2006). However, re-evaluation of early studies revealed design limitations, and *post hoc* analyses failed to validate improvements in the critical primary outcome measures of motor and sensory function (Coleman et al., 2000; Hurlbert, 2000; Short et al., 2000). The inconsistency in results plus systemic complications led surgical societies to downgrade the GC therapy recommendation from standard-of-care for medical practice (highest scientific validity) to an option (lowest validity) (Hurlbert and Hamilton, 2008; Hadley and Walters, 2013). Nevertheless, the use of GCs in treating spinal cord injuries continues (Eck et al., 2006; Schroeder et al., 2014). Mechanistic investigation is needed to resolve this controversial issue.

The lack of significant regeneration of the injured mammalian cord is attributed to spinal neuron loss, quiescence of spared cells, inflammation, and a non-permissive microenvironment for axon regrowth. Despite evidence that astroglia can support regeneration (Anderson et al., 2016), the scar formed by reactive astroglial proliferation is considered to be a barrier to axon regrowth and functional recovery (Tom et al., 2004; Busch and Silver, 2007; Barnabé-Heider et al., 2010). As such, the non-permissive nature of the mammalian injury site limits interpretation of potential positive and negative regulators in studies of cord regeneration.

Evaluating events in a regeneration-permissive vertebrate species holds potential to unmask differences in the signaling pathways that contribute to paralysis following mammalian spinal cord injury. The spinal cord of zebrafish, both larval and adult, regenerates functionally following complete transection. These models enable investigation of the molecular and cellular mechanisms that control neural repair (Becker et al., 1997; van Raamsdonk et al., 1998; Guo et al., 2011; Goldshmit et al., 2012; Briona and Dorsky, 2014). One difference in zebrafish vs. mammals after spinal cord injury is that ependymal radial glial cells begin to proliferate, elongate, and translocate to form bipolar bridges that span the transection lesion to support axon regeneration (Goldshmit et al., 2012; Mokalled et al., 2016). The ependymal cells also yield multipotent neural precursors that replenish lost neural cell types (Reimer et al., 2008, 2009; Briona and Dorsky, 2014).

Regeneration in the injured zebrafish CNS (brain in the adult [Kyritsis et al., 2012] and spinal cord in larval fish [Ohnmacht et al., 2016; Tsarouchas et al., 2018]) is reduced by GC administration, which suppresses immune cell infiltration and is noted to correlate with reduced radial glial proliferation and regeneration. The role of GCs in regulating CNS neurogenic niches is poorly defined, but GCs have been reported to attenuate hippocampal neurogenesis in mammals and regeneration-competence in the chick retina (Wong and Herbert, 2005; Fitzsimons et al., 2013; Gallina et al., 2014; Gallina et al., 2015).

Nuclear receptor subfamily 3, group C, member 1, is a prominent GC receptor common to mammals and zebrafish (Nr3c1 in fish; NR3C1 in mammals [Stolte et al., 2008; Alsop and Vijayan, 2009]). When ligated by GC, the cytoplasmic receptor enters the nucleus and serves as a transcription factor (Bridgham et al., 2006). Its binding to GC response element sequences in DNA stimulates transcription (Beato and Klug, 2000), particularly of genes that suppress inflammation (De Bosscher et al., 2008). In the adult rat spinal cord, *nr3c1* mRNA is distributed widely, and its expression increases within 4 h of cord injury (Yan et al., 1999). The effects of GCs on neural cells following cord injury are unknown.

Based on earlier studies in zebrafish and chicks, we hypothesized that GCs may be a key inhibitory factor for functional regeneration, (Kyritsis et al., 2012; Gallina et al., 2014, 2015; Ohnmacht et al., 2016; Tsarouchas et al., 2018). Our initial aim was to identify GC target cells that are pertinent to neural regeneration. While investigating functional spinal cord regeneration in the injured larval zebrafish, we discovered that GCs inhibit neural repair by stimulating Nr3c1 signaling in ependymal glia. Conversely, the signaling activity of this receptor in ependymal glia of adult rats was activated solely by cord transection. These complementary data plausibly explain the limited neural repair that occurs in the mammalian CNS, and further suggest that failure of the injured spinal cord to regenerate in a clinical context might be compounded by conventionally administered high-dose corticosteroid therapy.

## Materials and Methods

### Experimental Organisms

Wild-type stocks were derived from offspring of adult zebrafish purchased from Segrest Farms (Florida, United States). Studies were approved by the Mayo Clinic Institutional Animal Care and Use Committee (IACUC protocol A36315-15). Care of fish accorded with standard protocols. Adult and embryonic fish were maintained at 28.5°C on a 14-h light/10-h dark cycle. Larvae were raised in standard E2 embryo media, on a diet of paramecium from day 5 post-fertilization (dpf) with brine shrimp supplementation from 14–28 dpf (the sole diet thereafter).

Rats were housed per National Institutes of Health and United States Department of Agriculture guidelines on a 12 h light-dark cycle and standard food and water *ad libitum*. Each experimental group had six adult female Fischer rats (8–10 weeks old, weighing 180–200 g; female rats are easier to handle and less prone to urinary tract infection from bladder squeezing), or P0 neonatal rats of unknown sex for Supplemental Figure S1. Studies were approved in IACUC protocol A00001766-16.

### Zebrafish: Spinal Cord Injury, EdU and Dexamethasone Treatments

Tricaine methanesulfonate (Argent Chemical Laboratories, Redmond, WA, United States) was added to medium in 100 mm × 15 mm Petri dishes (Corning, Durham, NC, United States) as anesthetic for spinal manipulations; overdose was used for killing. The cord was transected following established protocols (Bhatt et al., 2004), with 3-dpf larval fish mounted on Sylgard silicone elastomer (Dow Corning, Midland, MI, United States) stages cast from microinjection molds (Adaptive Science Tools, Worcester, MA, United States). Borosilicate capillary tubing (World Precision Instruments, Sarasota, FL, United States) was pulled, broken and inserted through the cord under a Zeiss Discovery V12 stereoscope. Rare fish that bent along the body axis due to notochord damage were excluded from the study. Sham injured and uninjured clutchmate controls were anesthetized and exposed to the same intra-operative conditions as injury groups. In specified studies, the synthetic GCR agonist Dexamethasone (Sigma), or vehicle, was added to the holding bath (0.01% total volume methanol vehicle) immediately after injury. Solutions were replaced every 48 h. Consistent with published data (Hillegass et al., 2007; Wilson et al., 2013, 2015), our study of a dose range (1–100 μM) established 10 μM as the ideal GC concentration (minimal sufficient to inhibit functional recovery following cord transection). This dose did not affect behavioral responses, gross morphology, or survival in uninjured control fish exposed for 120 h.

### Rats: Spinal Cord Injury and Post-operative Care

Rats were monitored and cared for by veterinarians experienced in handling rodents with spinal cord injury. Ibuprofen was added to drinking water 40 mg/L beginning 48 h prior to surgery and thereafter. Rats were randomly assigned to experimental groups for T9 laminectomy alone or laminectomy followed by complete spinal cord transection, per protocols described in Chen et al. (2011). Anesthesia was induced via intraperitoneal ketamine (80 mg/kg; Fort Dodge Animal Health, Fort Dodge, IA, United States), xylazine (5 mg/kg; Lloyd Laboratories, Shenandoah, IA, United States) and maintained by inhaled isoflurane (1.5–2%; Cardinal Health, Dublin, OH, United States) as needed for surgery. Rats were kept on a 37°C heating pad during surgery and recovery from anesthesia. The spinal cord was cut with a sharp blade (No 11, BD Medical, Batavia, IL, United States), then probed with a microhook to confirm complete transection. Post-operatively rats were housed individually in low-walled cages, with soft chow provided on the cage floor. Following surgery, saline was injected intraperitoneally (5 mL) and, thereafter, bladders were squeezed twice daily. Urine was examined for signs of infection. Analgesics and antibiotics were given as needed.

### Rat Tissue Preparation

Adult rats were killed by injecting intraperitoneally 0.4 mL pentobarbital sodium (40 mg/kg; Fort Dodge Animal Health, Fort Dodge, IA, United States), and perfused via the heart with saline followed by 4% PFA (Chen et al., 2011). The lesioned spinal cord region and adjacent tissues were removed, and fixed for 72 h in PFA at 4°C. The spinal cord tissue was isolated with approximately 10 mm of adjacent tissue on each side, fixed overnight in 4% PFA at 4°C, and processed for cryosectioning. Neonatal (P0) rats were killed by decapitation. Approximately 10 mm of tissue on each side of the T9 vertebral level spinal cord and adjacent tissues were dissected, and fixed for 48 h in 4% PFA at 4°C.

### Zebrafish Whole-Mount Immunohistochemistry

Immediately after transecting injury, zebrafish were transferred to medium containing 0.5 mM ethynyldeoxyuridine (EdU). Killed fish were fixed overnight at 4°C, in either 9 parts 100% ethanol: 1 part 37% formaldehyde (for PCNA IgG labeling) or PBS containing 4% PFA and 5% sucrose, then stored in methanol (-20°C). After rehydrating with PBS containing 1% Triton X-100, and removing pigment with 1% H_2_O_2_ and 5% formamide diluted in PBS under intense light exposure, specimens were permeabilized using proteinase K. EdU uptake was detected by incubating for 2 h with Click-it EdU AF 594 kit following manufacturer’s protocol (Thermo Fisher Scientific). Following 2 h incubation at 25°C in PBS containing 10% normal goat serum, 2% DMSO and 1% Triton X-100 (blocking buffer), specimens were incubated for 16–48 h at 4°C, with gentle rocking, in blocking buffer containing primary antibodies: mouse anti-PCNA monoclonal IgG (1:500; clone PC10, Sigma-Aldrich, St. Louis, MO, United States; Cat# P8825, **RRID:AB_477413**), rabbit anti-EGFP polyclonal antiserum (1:250; Life Technologies, Eugene, OR, United States; Cat# A-6455, **RRID:AB_221570**), chicken anti-GFP polyclonal antiserum (1:500; Abcam, Cambridge, MA, United States; Cat# ab13970, **RRID:AB_300798**), mouse anti-acetylated tubulin monoclonal IgG (1:200; Sigma Aldrich; Cat# T7451, **RRID:AB_609894**), mouse anti-HuC/D monoclonal IgG (1:300; Life Technologies; Cat# A-21271, **RRID:AB_221448**). Specimens were washed in PBS containing 1% Triton X-100 and incubated overnight at 4°C in secondary antibody diluted 1:250. Nuclei were labeled with DAPI (1: 5000; Thermo Fisher Scientific). Secondary antibodies were: Alexa Fluor goat anti-primary IgG (488, 568, 594, and 647; Life Technologies). After washing in PBS containing 1% Triton X-100, specimens were mounted on coverglass with ProLong Diamond antifade reagent (Life Technologies).

### Cryopreservation and Cryosection Immunohistochemistry

Cryopreservation and immunohistochemical methods for rat and zebrafish tissues were identical. Zebrafish were fixed for 16 h at 4°C in 4% PFA or ethanol: formaldehyde (as above), washed in 5% sucrose/PBS and 30% sucrose/ PBS, incubated for 4 h in 2:1 O.C.T.: 30% sucrose/PBS, frozen (-20°C) in O.C.T. (Sakura Finetek, Torrance, CA, United States), then cryosectioned at 16 μm. Sections were dehydrated at 55°C for 2 h and stored at -80°C. Slides were thawed for 20 min at 55°C and rehydrated in PBS. For antigen retrieval, we used 10 mM sodium citrate (pH 6.0) with 0.1% Tween-20 (95°C, 15 min). Sections were incubated in PBS/4% normal goat or chicken serum/0.4% Triton X-100/1% DMSO blocking solution for 1 h at 25°C and 16 h at 4°C in primary antibody diluted in blocking buffer. Primary antibodies were: mouse anti-PCNA monoclonal IgG (1:1000, clone PC10, Sigma-Aldrich), rabbit anti-PCNA polyclonal antiserum (1:500, Abcam; Cat# ab19166, **RRID:AB_444779**), rabbit anti-EGFP polyclonal antiserum (1:750, Thermo Fisher Scientific), chicken anti-GFP polyclonal antiserum (1:250; Abcam), rabbit anti-GFAP polyclonal antiserum (1:500, Dako, Carpinteria, CA, United States; Cat# M0761, **RRID:AB_210995**), mouse anti-GFAP monoclonal IgG (1:250, Sigma-Aldrich; Cat# G3893, **RRID:AB_477010**), goat anti-Sox2 polyclonal antiserum (1:100, R&D Systems, Minneapolis, MN, United States; Cat# AF2018, **RRID:AB_355110**), chicken anti-vimentin antiserum (1:400. EMD Millipore), rabbit anti-Nr3c1 polyclonal IgG (1:50, Thermo Fischer Scientific, PA1-511A; Cat# AB5733, **RRID:AB_11212377**; characterized by Gallina et al., 2015). Washes were in 0.05% Tween-20/ PBS and incubation in secondary antibodies diluted in 0.05% Tween-20/ PBS for 1 h at 25°C. Secondary antibodies were: Alexa-Fluor-conjugated (488, 568, or 647) goat or chicken anti-primary IgG (1:500, Life Technologies, Eugene OR, United States). Nuclei were labeled with DAPI (1:15000; Thermo Fisher Scientific; **RRID:AB_2307445**). After repeated washes, sections were mounted with glass coverslips and Prolong Diamond Antifade Mountant (Life Technologies).

### TUNEL

Terminal deoxynucleotidyl transferase-mediated biotinylated UTP nick end labeling (TUNEL) was performed on 4% PFA fixed whole-mount zebrafish using the ApoAlert DNA Fragmentation Assay kit (Clontech, Mountain View, CA, United States). Pigment was removed by incubating in 1% H_2_O_2_ and 5% formamide in PBS under intense light exposure, followed by tissue permeabilization with Proteinase K. Enzymatic labeling of fragmented DNA, according to the manufacturer’s protocol in label buffer containing 2% biotinylated dUTP (Roche Diagnostics, Indianapolis, IN, United States). Samples were processed and dUTP was detected by Alexa-fluor conjugated Strepavidin (Life Technologies, Eugene, OR, United States).

### Behavioral Assays

To measure functional recovery, locomotor function was scored on a scale from 1 to 5 at designated time points. Locomotor recovery assay scores (Goldshmit et al., 2012) for larval fish were: (1) body paralyzed caudal to the lesion with fish lying on its side at the bottom of the dish, non-responsive to tail prod; (2) fish oriented upright, non-responsive to tail prod or responding with brief non-productive movement rostral to the lesion site; body paralyzed caudal to the lesion site; (3) tail prod evoked brief uncoordinated locomotion; (4) fish able to escape, with movements sustained for a longer period and becoming coordinated; (5) locomotion indistinguishable from sham-injured fish. Scores were obtained at times indicated, from the average values for each fish tested in triplicate (with periods of rest).

### TALENs

Targeted *nr3c1* mutation using transcription activator-like effector nucleases (TALENs) was performed as described in Krug et al. (2014). Briefly, one-cell embryos were microinjected with 50 pg of mRNA encoding *nr3c1*-targeting (ZFIN ID: ZDB-TALEN-150928-1) or GM2 (non-targeting) TALEN pairs. Mutation of *nr3c1* was confirmed by RT-PCR (not shown) and Nr3c1 immunostaining (see Supplementary Figure S2).

### Quantification and Statistical Analysis

**FIGURE 1:**
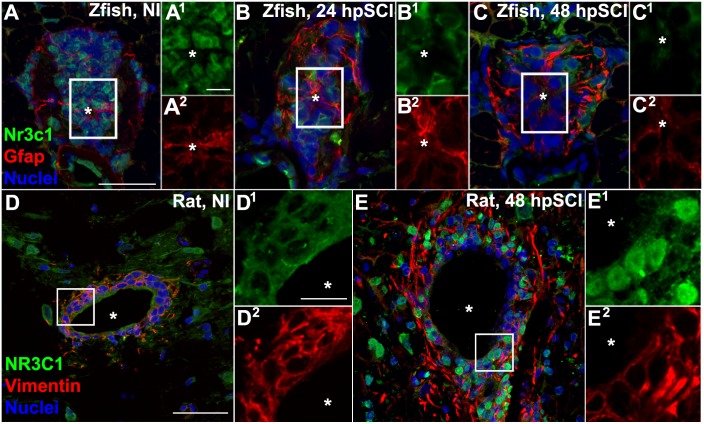
Spinal cord injury stimulates differential Nr3c1 expression by ependymal glia in zebrafish and rats. **(A–C)** Immunostaining for Nr3c1 (green) and Gfap (red), plus DAPI staining (blue) in larval zebrafish spinal cord transverse sections at 24 h after sham injury **(A)**, or 24 h **(B)**, and 48 h **(C)** post SCI. The nuclear Nr3c1 distribution in Gfap+-ependymal glia without injury **(A^1,2^)** is shifted to cytoplasmic at 24 and 48 h post SCI **(B^1,2^,C^1,2^)**. Scale bars, 20 μm (**A**, same as **B,C**) and 5 μm (**A^1^**, same as **A^2^–C^2^**). Boxed regions denote ependymal glial cell bodies around the central canal (^∗^) and are magnified 167% **(A^1,2^–C^1,2^)**. Representative images from 10 zebrafish (three sections per cord examined). See Supplementary Figure S2 for validation of Nr3c1 immunoreactivity. **(D,E)** Adult rat spinal cord transverse sections examined at 48 h post sham injury **(D)** and 48 h post SCI within 300 μm rostral of transection **(E)**. The cytoplasmic Nr3c1 expression in ependymal glia in controls **(D^1,2^)** is shifted to the nucleus after SCI **(E^1,2^)**. Scale bars, 50 μm (**D**, same as **E**) and 12.5 μm (**D^1^**, same as **D^2^–E^2^**). Boxed regions denote ependymal glial cell bodies around the central canal and are magnified 333% **(D^1,2^,E^1,2^)**. Representative images from six rat spinal cords (three sections per cord examined).

Microscopy was performed with an Olympus Fluoview FV1000 confocal microscope. Cell counts from lateral views of whole-mounted zebrafish (20-μm thick confocal *z*-stacks) were obtained at 100 μm from the center of the lesioned tissue. Additionally, 10-μm thick *z*-stacks were obtained from six consecutive spinal cord cross sections spanning the lesioned tissue where indicated in the figure legend (see Figure 5). Cell counts and quantification of colocalizations were performed manually with the ImageJ plugin Cell Counter. Six rats were examined in each group for Figure 1. For zebrafish experiments, 10 individuals were killed for spinal cord examination at each experimental time point and for each condition (numbers are reported in the figure legends). Images were cropped, high backgrounds were reduced and low-intensity signals were enhanced in Adobe Photoshop (Adobe Systems, San Jose, CA, United States). Fluorescence levels were modified identically in all layers within a panel and in all other panels in a figure. Statistical analysis of the data was calculated with Student’s *t-*test (two-tailed and two-sample unequal variance) of pair-wise comparisons between the groups in Excel software. Actual *p*- and *n*-values are listed in the text; *p* < 0.05 and < 0.01 were considered, respectively, significant (^∗^) and highly significant (^∗∗^). Axon regeneration was quantified by corrected total fluorescence measurements. Integrated density calculations of immunofluorescence were obtained from spinal cord tissues within 50 μm of the lesion center using ImageJ software. Measurements for each image were normalized to regions within the same field of view containing background signal. Corrected total fluorescence values were obtained and plotted using Excel software by subtracting the mean integrated density of background measurements from the fluorescence measured in the tissue region of spinal cord injury (Burgess et al., 2010).

## Results

### Ependymal Glial Glucocorticoid Receptor (Nr3c1) Is Regulated in Opposite Directions After Transecting Spinal Cord Injury in Zebrafish and Rat

Loss of the inhibitory signals that normally constrain regeneration-competent cells in a latent state in zebrafish may contribute to functional repair of the injured spinal cord in that species. We hypothesized that GCs may be a key inhibitory factor, based on earlier regeneration studies in zebrafish and chick (Kyritsis et al., 2012; Gallina et al., 2014, 2015; Ohnmacht et al., 2016; Tsarouchas et al., 2018). We aimed therefore to identify and compare the cell types targeted by GCs after spinal cord injury in the robustly regenerative larval zebrafish and the non-permissive milieu of adult rats.

To identify the cellular targets of GCs and investigate the potential role of Nr3c1 in neural repair, we evaluated Nr3c1 immunostaining, following established protocols (Gallina et al., 2014, **RRID:AB_10742538**), in tissue adjacent to a cord lesion (Figure 1A–C). In uninjured control fish, Nr3c1 immunoreactivity was strong in nuclei of ependymal glia surrounding the central canal (positive for both glial fibrillary acidic protein [Gfap], **RRID:AB_210995** and DAPI, **RRID:AB_2307445**) (Figure 1A). Nuclear localization is consistent with constitutive Nr3c1 stimulation. The Gfap-negative cells that expressed nuclear Nr3c1 were neurons (HuC/D immunoreactive, **RRID:AB_221448**; not shown). The intensity of Nr3c1 immunoreactivity was reduced following spinal cord injury. By 24 h, Nr3c1 in ependymal glia was restricted to the cytoplasm; at 48 h, immunoreactivity was further reduced (Figure 1B,C). The reduced expression and cytoplasmic redistribution of Nr3c1 suggested post-injury loss of Nr3c1 signaling. These dynamic changes in Nr3c1 expression implicated ependymal glia as a potential GC target.

Rat *nr3c1* mRNA is expressed widely (Yan et al., 1999), but little is known of the receptor’s activity in neural cells in the injured spinal cord. Comparative immunohistochemical staining of sham-injured adult rat spinal cord revealed cytoplasmic co-expression of NR3C1 and vimentin (**RRID:AB_11212377**) in ependymal glia surrounding the central canal (Figure 1D,E). By 48 h post-transection, NR3C1 immunoreactivity in ependymal glia was strikingly redistributed from the cytoplasm to the nuclear compartment, consistent with receptor activation. Translocation of NR3C1 in ependymal glia implicates GC receptor signaling in these neural cells. Examination of neonatal (P0) rat spinal cords verified that ependymal glia had only basal NR3C1 expression in the cytoplasm (Supplementary Figure S1). This evidence supports the conclusion that the differential regulation of NR3C1 that we observed was due to species-intrinsic differences. Thus, following spinal cord injury, NR3C1 distribution and expression level are regulated in opposite directions in ependymal glia of rats compared to zebrafish.

### Exogenous Glucocorticoids BlockFunctional Recovery From Spinal CordInjury via Nr3c1 Activation

**FIGURE 2:**
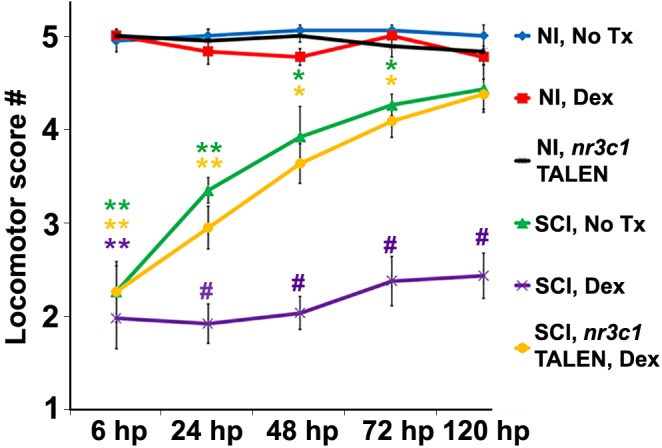
Glucocorticoids inhibit functional recovery following SCI. Locomotor recovery score of wild-type or *nr3c1* targeting TALEN-injected zebrafish that received no treatment or Dex measured at 6, 24, 48, 72, and 120 h post sham injury or SCI. Locomotor responses were quantified from uninjured controls with no treatment controls (blue line), Dex (red line), and *nr3c1* TALEN injections (black line), or from SCI with no treatment (green line), Dex (purple line), and Dex with *nr3c1* TALEN injections combined (yellow line). Data show the mean ± SEM; *n* = 10 per time point in each condition (^∗^*p* < 0.05; ^∗∗^*p* < 0.01 [compared to uninjured controls], and ^#^*p* < 0.01 [compared to both uninjured controls and SCI no treatment]). ^∗^ and ^#^ color coded to match corresponding data points.

Finding that Nr3c1 expression in ependymal radial glia following spinal cord injury is regulated differentially in zebrafish and rats led us to investigate whether GC pathway activation might be sufficient to block functional regeneration. Functional recovery from injury is robust in larval zebrafish at 120 h after cord transection (Bhatt et al., 2004; Briona and Dorsky, 2014). Evoked swimming activity depends on motor responses; its measurement after complete spinal cord transection enabled quantitation of locomotor recovery (Goldshmit et al., 2012). Compared to uninjured control fish, responses were severely impaired at 6 h, but had improved by 24 and 48 h after transection (Figure 2). Swimming behavior was robust by 72 h and nearly indistinguishable from that of uninjured control groups by 120 h. In contrast, behavioral responses were significantly impaired in fish treated with the synthetic GC Dexamethasone (Dex, 10 μM; *p* < 0.01), but not in uninjured Dex recipients (*p* = 0.15). We next downregulated *nr3c1* expression by customized genome editing (TALENs). Analysis by RT-PCR confirmed *nr3c1* mutation in embryos injected with mRNAs encoding targeting TALENs (Krug et al., 2014; not shown). Targeted loss of Nr3c1 immunoreactivity in *nr3c1* TALEN-injected fish verified the Nr3c1 antibody’s specificity. Expression was reduced by comparison with non-targeting GM2 TALEN-injected controls (Supplementary Figure S2). Behavioral recovery following spinal cord injury and Dex treatment in fish with mutated *nr3c1* did not differ significantly from controls at 120 h post spinal cord injury (*p* = 0.85). Furthermore, the *nr3c1* TALEN-injected zebrafish controls (no injury, no Dex) had no behavioral phenotype compared to non-transected controls (*p* = 0.36). Thus, inhibition of locomotor recovery from spinal cord injury by the synthetic GC, Dex, is Nr3c1-dependent and not due to an off-target effect.

To evaluate the extent of cell death following spinal cord transection, and to determine whether or not Dex altered cell viability (a potential reason for decreased locomotor recovery), we utilized TUNEL assays to evaluate DNA fragmentation in zebrafish transected cords, both Dex-treated and controls. Counts of TUNEL-positive nuclei in the spinal cord domain of whole-mount preparations (from *z*-stacks at 100 μm from the lesion center) showed no significant change in the number of cells dying at the peak of injury-induced apoptosis in Dex–treated fish (*p* = 0.3; Supplementary Figure S3). We concluded from these findings that impairment of locomotor recovery in Dex recipients is attributable to regulation of a critical aspect of regeneration rather than to altered cell viability.

### Glucocorticoids Inhibit Ependymal Glial Support of Regeneration

**FIGURE 3:**
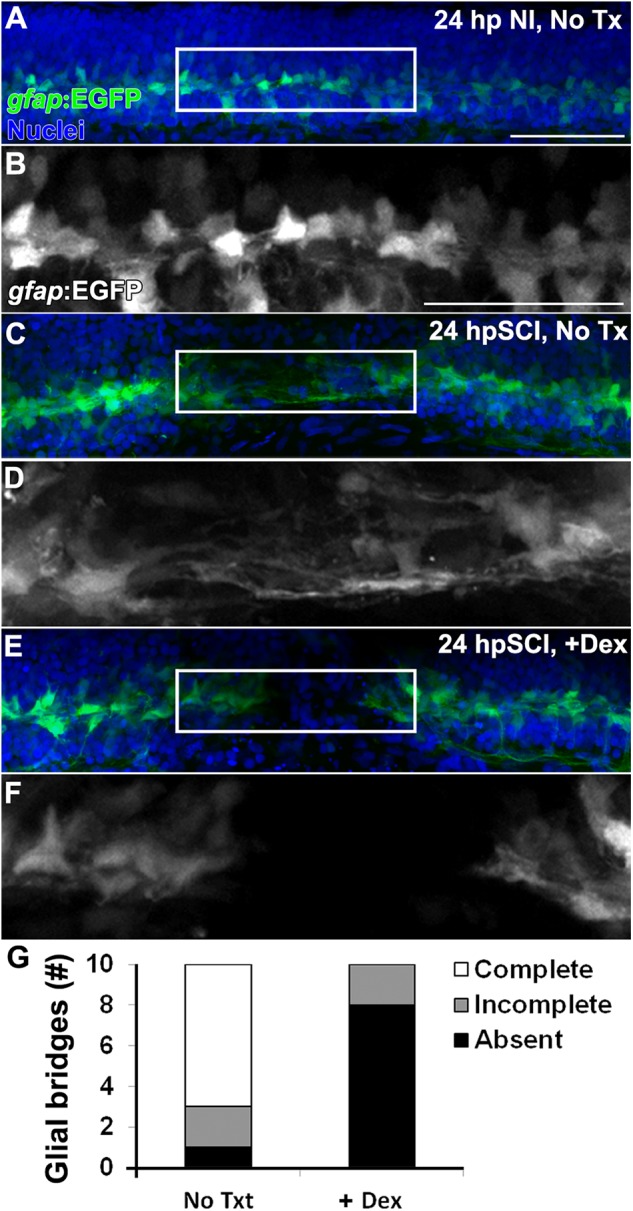
Glucocorticoids inhibit the formation of *trans*-lesion glial bridges. **(A–F)** Whole-mount preparations (lateral view) of Tg(*gfap*:EGFP) transgenic zebrafish show immunostaining for EGFP (green) and DAPI staining (blue) from 24 h post sham injury **(A,B)**, 24 h post SCI with no treatment **(C,D)** and 24 h post SCI+Dex **(E,F)**. EGFP-positive ependymal glia were observed spanning the lesion site at 24 h post SCI in controls **(C,D)**, and were absent with Dex **(E,F)**. Boxed regions **(A,C,E)** are magnified by 175% (**B,D,F**, respectively). Scale bars, 50 μm (**A** is the same as **C,E**; **B** is the same as **D,F**). Representative images from 10 preparations per condition. **(G)** Counts of glial bridges at 24 h post SCI in and 24 h post SCI+Dex.

Based on the observed dynamics of Nr3c1 expression in the zebrafish spinal cord (Figure 1), we hypothesized that GCs inhibit functional recovery by impairing the capacity of ependymal glia to support regeneration. Morphologically, ependymal glia in the transected zebrafish cord form elongated bipolar bridges that span the lesion site and correlate with *trans*-lesional axon regeneration (Goldshmit et al., 2012; Mokalled et al., 2016). To investigate whether or not GCs regulate *trans*-lesional glial bridge formation following cord injury, we evaluated transgenic reporter fish, Tg(*gfap*:EGFP), which express EGFP in radial glia (Kassen et al., 2007; ZFIN ID: ZDB-ALT-070410-12), and compared Dex-treated to controls. Ependymal glia in uninjured control cords paralleled the central canal (Figure 3A,B). At 24 h after injury (Figure 3C,D,G), elongated processes of ependymal glia completely spanned the lesion (7 of 10) or partially extended into the injury site (2 of 10). However, with Dex treatment, the rostral and caudal cord remained separated, and bipolar glial bridges were absent (8 of 10) or incomplete (2 of 10; Figure 3E–G). Thus, within 24 h of transection, ependymal glia in the injured larval spinal cord become bipolar. The blockade of *trans*-lesional glial bridges by Dex treatment establishes an inhibitory role for GCs.

**FIGURE 4:**
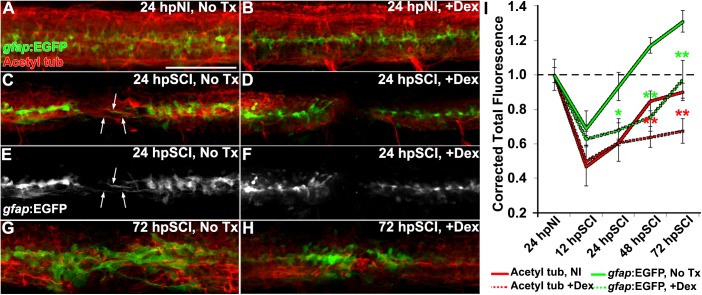
Glucocorticoids suppress glial bridges and axon regrowth. **(A–H)** Whole-mounted Tg(*gfap*:EGFP) zebrafish (lateral view) show spinal cord immunostaining for EGFP (green) and acetylated tubulin (axons, red) in uninjured controls **(A,B)**, SCI followed by no treatment **(C,E,G)**, and SCI with Dex treatment **(D,F,H)** for 24 and 72 h post SCI. Arrows indicate bipolar EGFP-positive glial bridges **(C,E)** and juxtaposed axons **(C)** that have entered the lesion at 24 h post SCI, but are absent with Dex treatments **(D,F)**. Scale bar, 50 μm **(A–H)**. **(I)** Quantification of the mean corrected total fluorescence intensity for axons (red lines) and ependymal glia (green lines) within 50 μm of the lesion center from no treatment controls (solid lines) and +Dex (dashed lines) relative to uninjured controls. Data show means ± SEM. ^∗^*p* < 0.05; ^∗∗^*p* < 0.01 compared to SCI no treatment; *n* = 10 zebrafish per time point in each condition. ^∗^ and ^∗∗^ are color coded to match corresponding data points.

We next investigated whether GCs might modulate axon regrowth in the context of morphological changes in ependymal glia of Dex-treated Tg(*gfap*:EGFP) zebrafish (Figure 4). Acetylated tubulin immunoreactivity (**RRID:AB_609894**) identified axons in whole-mount preparations. We measured the fluorescence levels of axons and ependymal glia (from maximal projection *z*-stacks within 50 μm of the lesion center [Figure 4I]) in the spinal cord domain of untreated controls (Figure 4A,C,E,G), and compared to Dex-recipients (Figure 4B,D,F,H). Cords of sham-injured fish were densely populated with axons and ependymal glia (Figure 4A,B, respectively). At 12 h after injury, the earliest time point studied, the fluorescence intensity was reduced significantly in both axons and ependymal glia (Figure 4I, *p* < 0.01). At 24 h (Figure 4C,E), axons within the lesion were closely associated with bipolar glia (Figure 4E,F, arrows). At 48 h, the fluorescence intensity of axons and ependymal glia had increased (Figure 4I). By 72 h, the fluorescence intensity of axons approached pre-injury levels, rostral and caudal sides of the cord were rejoined, and the ependymal glial domain was expanded at the lesion site (Figure 4G,I). By contrast, the fluorescence intensity of axons in Dex recipients was reduced at 24 h post cord transection and bipolar glial activity was inhibited (Figure 4D,F,I, *p* = 0.01). At 48 h and 72 h, the fluorescence intensity in Dex recipients remained significantly attenuated (Figure 4H,I; *p* < 0.01). Axonal extension into the lesion site was not observed in the absence of bipolar glia. Thus, regeneration was initiated within 24 h of cord injury, axons were observed associated with bipolar glia spanning the lesion site and, by 72 h, acetylated tubulin imunoreactivity within the lesion was restored to the pre-injury state. Dex inhibited the formation of bipolar ependymal glial as well as subsequent axon regrowth. These findings are consistent with earlier demonstrations that ependymal glial bridges facilitate axon regrowth across cord lesions (Goldshmit et al., 2012; Mokalled et al., 2016).

**FIGURE 5:**
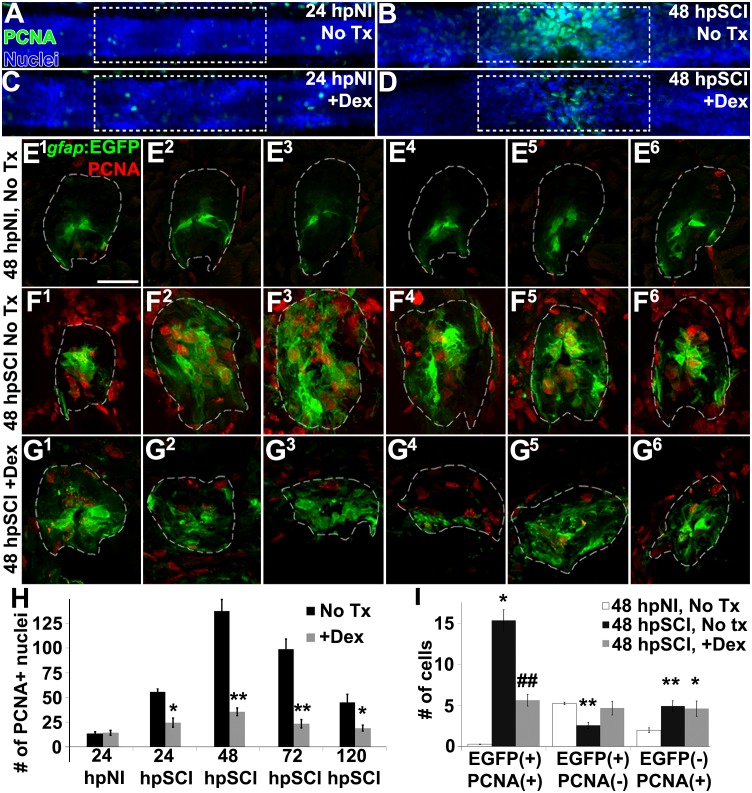
Glucocorticoids inhibit ependymal glial proliferation following SCI. **(A–D)** Whole-mounted larval zebrafish show immunostaining for PCNA (green) and DAPI staining (blue) in the spinal cord after sham injury **(A,C)** and 48 h post SCI **(B,D)**, either followed by no treatment **(A,B)** or Dex treatment **(C,D)**. Dashed boxes represent a 200-μm region centered at the lesion site used for quantifying cell counts and serve as scale bars (**A** is the same as **B–D**). **(E–G)** Spinal cord transverse sections in Tg(*gfap*:EGFP) zebrafish were immunostained to detect PCNA (red) and EGFP in six concurrent 16-μm thick cryosections that spanned the lesion site at 48 h post sham injury **(E^1^–E^6^)**, 48 h post SCI followed by no treatment **(F^1^–F^6^)**, and 48 h post SCI + Dex **(G^1^–G^6^)**. Scale bar, 20 μm **(E–G)**. Dashed outlines define the spinal cord area (as determined by DAPI staining [DAPI not shown to highlight colocalization]) used for quantifying cell counts. **(H)** Mean number of PCNA-positive cells ± SEM quantified from within a 100-μm region centered on the lesion (dashed boxes in **A–D**). ^∗^*p* < 0.05; ^∗∗^*p* < 0.01; *n* = 10 zebrafish per time point in each condition. Comparisons were to the no treatment group for each time point. **(I)** Mean number of PCNA-positive and EGFP-positive cells ± SEM quantified within spinal cord (dashed outlines in **E–G**) cross sections in uninjured controls, SCI without treatment, and SCI + Dex. ^∗^*p* < 0.05; ^∗∗^*p* < 0.01 compared to sham injury; ^#^*p* < 0.05 and ^##^*p* < 0.01 compared to both sham and SCI control groups; *n* = 10 per time point in each condition.

To evaluate the potential role of GCs in regulating cell division following spinal cord injury, we counted proliferating cells (PCNA-positive nuclei, **RRID:AB_477413**) from *z*-stacks within the spinal cord domain, at 100 μm from the lesion center, comparing Dex recipients to controls (Figure 5A–D,H). Dex did not alter proliferation significantly in sham-injured controls (average PCNA-positive nuclei per spinal cord: 12.6 ± 2.4 and 13.8. ± 2.6, respectively, *p* = 0.73). Proliferation had increased by 24 h after transection (55.6 ± 11.8), was maximal at 48 h (137.6 ± 10.2), and then fell progressively (99.1 ± 8.3 at 72 h; 45.0 ± 6.9 at 120 h). By contrast, proliferating cells in Dex-treated fish were significantly fewer at all time points examined after cord transection (24 h, *p* = 0.04; 48 h, *p* < 0.01; 72 h, *p* < 0.01; 120 h, *p* = 0.03). These data are consistent with GCs being inhibitory to cell proliferation in injured spinal tissue.

To identify the proliferating cell type(s) inhibited by GCs, we evaluated transgenic reporter fish expressing EGFP in radial glia (Tg[*gfap*:EGFP]; Figure 5E1–G6). We compared numbers of EGFP-positive glia and EGFP-negative non-ependymal cells that colocalized with PCNA-positive nuclei in six consecutive cross-sections spanning the cord lesion. The spinal cord domain was identified by DAPI staining (Figure 5E1–G6; dashed lines outline spinal cord domain; DAPI is not shown) As anticipated, the average number of proliferating ependymal glia was significantly greater in regions of transecting injury (15.6 ± 3.3) than in corresponding regions of uninjured controls (0.3 ± 0.2, *p* < 0.01); this number was significantly reduced in lesional regions of fish treated with Dex (5.5 ± 1.8, *p* < 0.01). Quiescent ependymal glial numbers were significantly less than in uninjured controls (2.6 ± 1.0 compared to 5.3 ± 0.4, *p* < 0.01) but numbers in Dex-treated groups with spinal cord injury were not significantly altered (4.6 ± 2.2, *p* = 0.55). Dividing non-ependymal cell numbers also were increased (4.3 ± 0.7) compared to uninjured controls (1.9 ± 0.7, *p* < 0.01), but that population (likely hematogenous cells, microglia and fibroblast-like cells [Wehner et al., 2017]) was not altered significantly by Dex treatment (4.9 ± 2.3, *p* = 0.80). Thus, approximately 75% of dividing cells were EGFP-positive ependymal glia or daughter neural precursor cells, and their proliferation post-injury was significantly reduced by Dex. These data agree with results reported previously from adult zebrafish studies, namely that Tg(*gfap*:EGFP) expressing ependymal glia and their daughter neural precursor cells (Reimer et al., 2008, 2009; Briona and Dorsky, 2014) are the predominant dividing cell types in injured spinal cord. We conclude from these findings that GCs inhibit ependymal glial proliferation.

**FIGURE 6:**
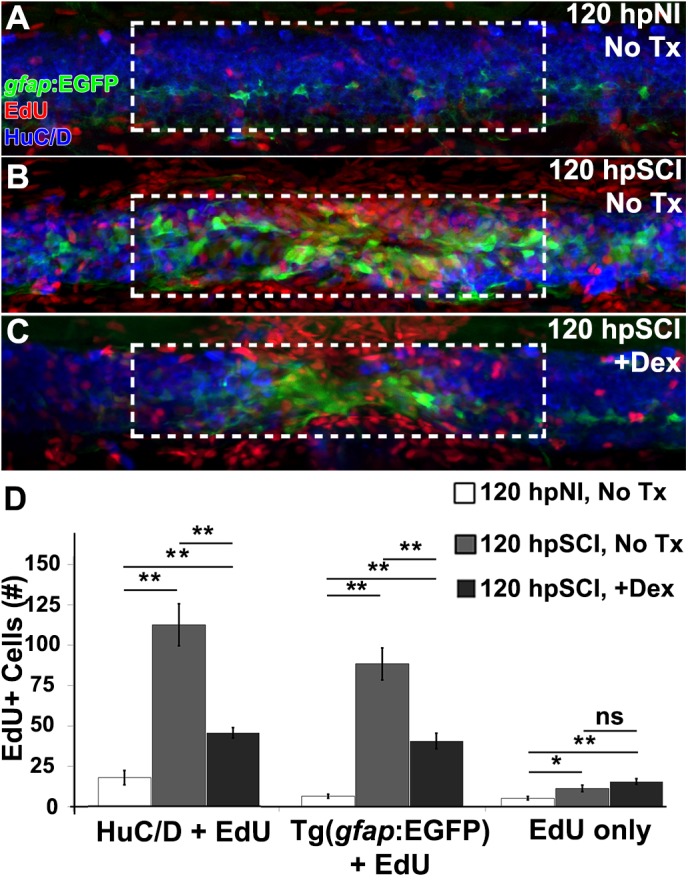
Glucocorticoids suppress neurogenesis. **(A–C)** Whole-mount preparations (lateral view) of Tg(*gfap*:EGFP) transgenic zebrafish show immunostaining for EGFP (green) and HuC/D (neurons, blue) along with detection of EdU (red) from 120 h post EdU incubation and sham injury **(A)**, SCI followed by no treatment **(B)**, and +Dex **(C)**. Dashed boxes represent a 200-μm region centered at the lesion site used for quantifying cell counts and serve as scale bars (**A** is the same as **B,C**). **(D)** Quantification of the mean number of EdU-positive cells within the spinal cord (± SEM). ^∗^*p* < 0.05; ^∗∗^*p* < 0.01; ns, not significant; 10 whole mounts per condition.

We next assessed, by EdU uptake, the regeneration of neurons and glia in Tg(*gfap*:EGFP) zebrafish treated with Dex (Figure 6). EdU-positive cells, identified as EGFP+ (glia), HuC/D+ (neurons) or cells of unknown lineage, were quantified from *z*-stacks within the spinal cord domain at ∼100 μm from the lesion center (Figure 6A–D). EdU-positive glial cell numbers were significantly increased at 120 h post-transection by comparison to sham-injured fish (*p* < 0.01). EdU-positive ependymal glia were significantly fewer after injury in Dex recipients (*p* < 0.01). Similarly, numbers of EdU-positive neurons were significantly increased after injury (*p* < 0.01), and significantly decreased after injury in Dex recipients (*p* < 0.01). Numbers of non-identifiable EdU-positive cells (likely hematogenous cells, microglia and fibroblast-like cells [Wehner et al., 2017]) significantly increased following injury (*p* = 0.02), but did not change significantly with Dex treatment (*p* = 0.11). In sum, our data demonstrate Dex attenuates neurogenesis following spinal cord injury by inhibiting proliferation of the ependymal glial source of new neural cells (Figure 5).

### Ependymal Glia Are Targets of Glucocorticoids in Spinal Cord Injury

**FIGURE 7:**
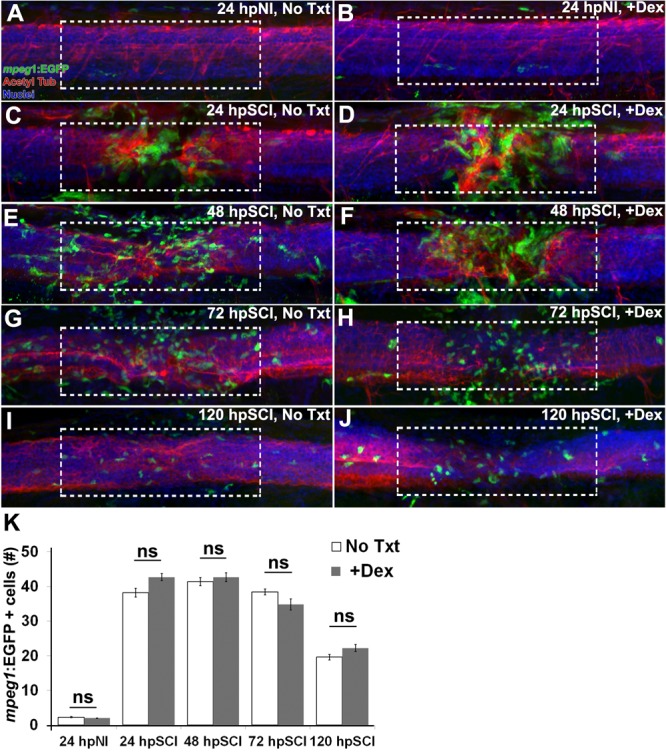
Responses of hematogenous cells and microglia to SCI and Dex treatments. **(A–J)** Whole-mount preparations (lateral view) of Tg(*mpeg1*:EGFP) transgenic zebrafish show immunostaining for EGFP (green) and acetylated tubulin (red), together with DAPI (blue) at 24 h post sham injury **(A,B)**, 24 h **(C,D)**, 48 h **(E,F)**, 72 h **(G,H)**, and 120 h **(I,J)** post SCI with no treatment **(A,C,E,G,I)**, and +Dex **(B,D,F,H,J)**. Dashed boxes represent a 200-μm region centered at the lesion site used for quantifying cell counts within the spinal cord domain and serve as scale bars (**A** is the same as **B–J**). **(K)** Quantification of the mean number of EGFP-positive cells within the spinal cord domain (± SEM). ns, not significant; 10 whole mounts per time point in each condition.

Repair of the injured adult zebrafish brain has been noted to correlate positively with the presence of microglia and hematogenous cells (Kyritsis et al., 2012), and attenuation of injured zebrafish spinal cord regeneration by high dose Dex (500 μM) is attributed to Dex suppression of those cells (Ohnmacht et al., 2016; Tsarouchas et al., 2018). To analyze the effect of the minimal dose of Dex that was sufficient to block regeneration in our study (10 μM), we used the transgenic reporter fish Tg(*mpeg1*:EGFP) (Ellet et al., 2011; ZDB-TGCONSTRCT-120117-1) to observe myeloid-derived cells infiltrating the lesioned spinal cord domain following complete transection (Figure 7). Numbers of EGFP-positive (green) cells in whole-mount preparations of control fish were compared with fish treated with Dex, from *z*-stacks within the spinal cord domain at 100 μm from the lesion center. Counterstaining with DAPI (blue) and acetylated tubulin immunostaining of axons (red) identified the spinal cord domain. At 24 h, uninjured controls contained 2.3 ± 1.3 EGFP-positive cells, not significantly different from uninjured Dex recipients (2.0 ± 0.9; *p* = 0.6). The number of EGFP-positive cells in injured cord did not decrease significantly regardless of Dex treatment (38.2 ± 1.2 and 42.7 ± 9.8 at 24 h [*p* = 0.5]. These cell numbers remained elevated at 48 and 72 h (41.4 ± 11.3 and 42.7 ± 12.7 [*p* = 0.8]; 38.4 ± 8.3 and 34.8 ± 15.6 [*p* = 0.6], respectively), and were declining at 120 h post-injury (19.6 ± 7.9 and 22.2 ± 10.0; *p* = 0.6). These findings suggest that low dose Dex suppresses neural repair by a mechanism independent of the immunosuppressive effects observed with higher Dex doses (Ohnmacht et al., 2016).

**FIGURE 8:**
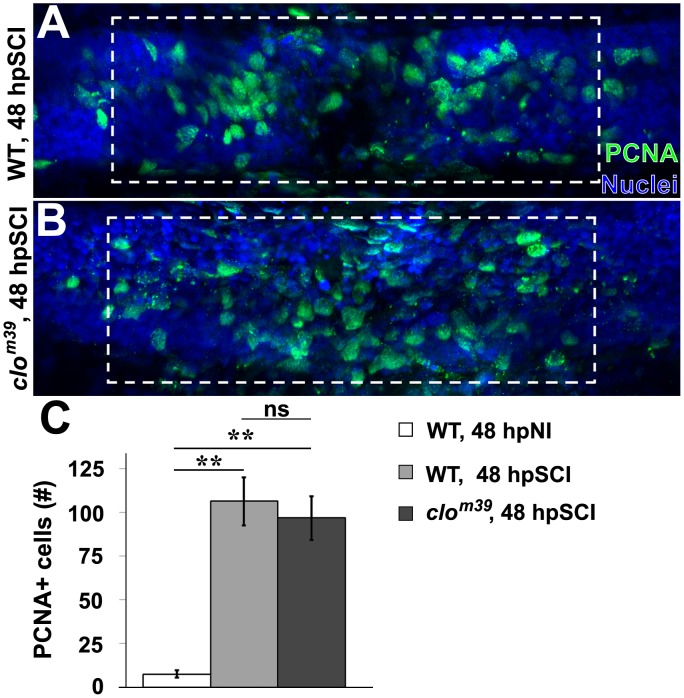
SCI-induced proliferation occurs without hematogenic or microglial responses. **(A,B)** Whole-mount preparations (lateral view) show spinal cords of wild-type **(A)** and *cloche* mutant **(B)** zebrafish immunostained for PCNA (green) at 48 h post SCI. Dashed boxes represent a 200-μm region centered at the lesion site used for quantifying cell counts and serve as scale bars (**A** is the same as **B**). **(C)** Quantification of PCNA-positive nuclei within the spinal cord domain **(A,B)**. Data show the means ± SEM (^∗∗^*p* < 0.01; 10 zebrafish per group).

To investigate the requirement of signaling molecules derived from hematogenous cells and microglia for initiating regeneration, we utilized the bloodless *cloche* mutant zebrafish (*clo*^m39^; Stainier et al., 1995). The *cloche* mutation affects a very early developmental stage, upstream of genes required for hematopoiesis and vasculature formation (Stainier et al., 1995; Liao et al., 1998; Lieschke et al., 2002; Qian et al., 2005; Bukrinsky et al., 2009; Reischauer et al., 2016). This allows larval spinal cord regeneration to be investigated in an immune-deficient background (Hasegawa et al., 2015). PCNA-positive nuclei were quantified in whole-mount preparations of homozygous *clo*^m39^ and wild-type zebrafish within the spinal cord domain, at ∼100 μm from the lesion center (Figure 8). At 48 h, the peak of ependymal glial proliferation (Figure 5), injured *clo*^m39^ spinal cords contained significantly more PCNA-positive nuclei (96.8 ± 12.6) than uninjured cords (7.5 ± 2.0, *p* < 0.01), but numbers did not differ significantly from transected wild-type cords (106.5 ± 4.6, *p* = 0.61). The modest reduction in numbers of proliferating cells observed in *clo*^m39^ spinal cords likely reflects the absence of microglia and hematogenous cells. These results suggest that neural cells are sufficient to initiate spinal cord regeneration.

**FIGURE 9:**
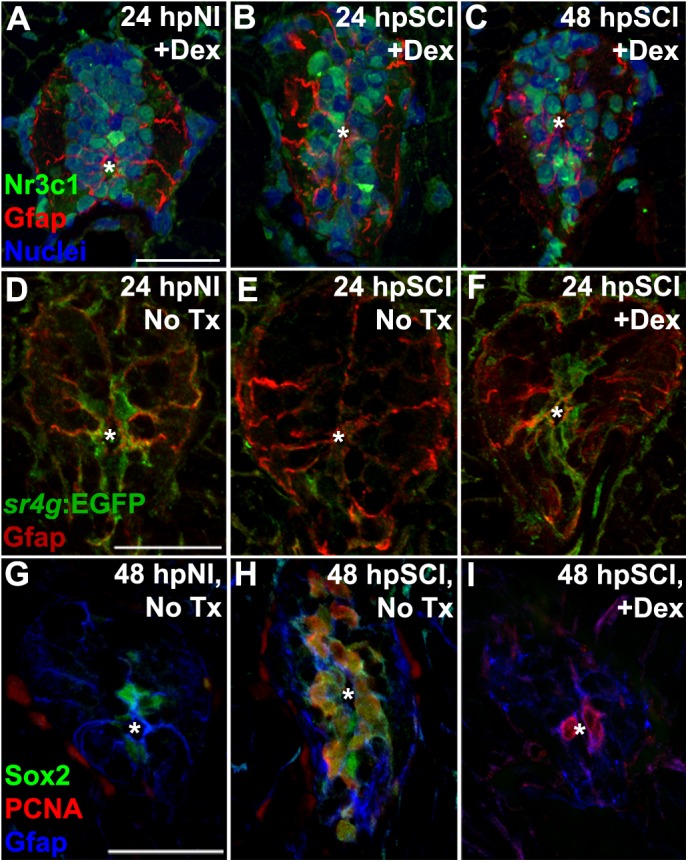
Direct effects of glucocorticoids in ependymal glia. **(A–I)** Zebrafish spinal cord transverse sections. **(A–C)** Immunostaining of wild-types for Nr3c1 (green) and Gfap (red), plus DAPI staining (blue) with Dex treatment at 24 h post sham injury **(A)**, 24 h **(B)** and 48 h post SCI **(C)**. Nuclear Nr3c1 (active) localization in Gfap-positive cells around the central canal is stimulated with Dex treatment. **(D–F)** Immunostaining for EGFP (pan-cytoplasmic) in the SR4G transgenic reporter line (green) and filamentous Gfap (red) at 24 h post sham injury **(D)** and SCI with no treatment **(E)** or 24 h post SCI +Dex **(F)**. Nr3c1 signaling is constitutively active in ependymal glia in uninjured controls **(D)**, diminished after SCI **(E)**, and was maintained after SCI + Dex. **(G–I)** Immunostaining for Sox2 (green), PCNA (red) and Gfap (blue) from 48 h post sham injury **(G)**, 48 h post SCI followed by no treatment **(H)**, or 48 h post SCI +Dex **(I)**. Sox2 immunoreactivity is reduced in ependymal glia and neural precursor cells after Dex treatment. Representative images from 10 spinal cords per time point in each condition. Scale bars, 20 μm (**A** is the same as **B,C**; **D** is the same as **E,F**; **G** is the same as **H,I**). ^∗^denotes central canal.

To determine if GCs directly affect ependymal glia, we investigated Nr3c1 after exposure to Dex (Figure 9). Similar to uninjured controls (Figure 1), Nr3c1 was localized to the nucleus of Gfap-positive ependymal glia in uninjured fish receiving 24 h Dex-treatment (Figure 9A). At 24 and 48 h after spinal cord transection and Dex treatment, Nr3c1 remained highly expressed and localized in the nucleus of ependymal glia (Figure 9B,C), consistent with elevated receptor activity. The persistence of nuclear Nr3c1 in ependymal glia after a transecting cord injury demonstrates that GCs target these cells.

We further identified the GC-targeted cell-types relevant to repairing the transected spinal cord by employing the SR4G transgenic zebrafish line to report Nr3c1 signaling activity with EGFP fluorescence (Krug et al., 2014; ZFIN ID: ZDB-FISH-151006-4). In uninjured control tissue, GC receptor signaling (green) was restricted to ependymal glia surrounding the central canal (Gfap-positive; Figure 9D, red). In agreement with our Nr3c1 analyses, 24 h after cord transection the GC receptor activation signal (EGFP intensity) in non-Dex-treated fish was reduced in ependymal glia within juxta-lesional cord cross sections (Figure 9E). However, the signal intensity remained robust and was restricted to ependymal glia in transected cord tissue of Dex recipients (Figure 9F). These observations support our hypothesis that GCs are an inhibitory cue for cord regeneration after transection. In contrast to previous studies (Kyritsis et al., 2012; Ohnmacht et al., 2016; Tsarouchas et al., 2018), our findings establish a new paradigm that GCs are inhibitory to spinal regeneration by stimulating Nr3c1 signaling in ependymal glia.

Suppression of injury-induced factors critical for neural repair is a plausible mechanism for GC regulation of ependymal glia. The transcription factor sex-determining region Y-box 2 (Sox2), an essential component of spinal cord regeneration, is highly expressed by zebrafish ependymal glia following injury. Sox2 is required for maximal proliferation of ependymal glia and their daughter neural precursor cells and for neurogenesis (Hui et al., 2014, 2015; Ogai et al., 2015). To determine if GCs regulate Sox2, we evaluated Sox2 immunoreactivity in ependymal glia in which a high level of GC receptor signaling was maintained by administering Dex (**RRID:AB_355110**; Figure 9G–I). In agreement with previous studies, Sox2 was expressed in ependymal glia of uninjured controls (Figure 9G). After transecting injury, Sox2 was highly expressed in ependymal glia and daughter neural precursor cells at the peak of proliferation (48 h after transection). Sox2 expression was reduced in both quiescent and dividing ependymal glia of Dex recipients. These results indicate that GC receptor signaling is a negative regulator of Sox2 expression, and inhibits spinal cord regeneration at the level of ependymal glia.

## Discussion

Our study has demonstrated that GC receptor signaling in ependymal glia is a critical negative regulator of functional regeneration in the injured spinal cord. By targeting ependymal glia directly, the GC receptor agonist, Dex, blocks functional recovery. Dex administration limits the formation of ependymal glial bridges, axon regeneration, ependymal glial proliferation, and neurogenesis. The apparent inverse regulation of ependymal glia by GCs in mammals and zebrafish plausibly explains why regeneration of the mammalian spinal cord is limited following injury.

Glucocorticoid receptors are expressed widely in the CNS of both larval zebrafish and adult rats (Yan et al., 1999). The receptor’s nuclear localization in ependymal glia of uninjured control fish indicates intrinsic signaling activity, while its cytoplasmic localization in the uninjured control cord of rats suggests low constitutive activity. The localization changes that we documented following spinal cord injury in the two species are consistent with differential regulation of signaling activity of the receptor, becoming cytoplasmic (inactive) in zebrafish and nuclear (active) in rats. The physiological relevance of ependymal glia as target of GCs was confirmed in our study of the SR4G transgenic zebrafish (reporter of GC receptor activity); signaling was constitutively active and restricted to ependymal glia in the non-perturbed spinal cord, but was silent 24 h after injury, coinciding with the onset of cell proliferation and bridge formation. However, when Dex was administered after cord transection, GC receptors remained nuclear and signaling persisted. The reduction of Sox2 expression in both ependymal glia and daughter cells following zebrafish cord transection further supports our conclusion that ependymal glia are a direct target of GCs, and identifies GC receptor activity in ependymal glia as an early determinant of functional regeneration.

The prominent inflammatory cell infiltration in the early post-injury phase (Martin and Feng, 2009; Loane and Byrnes, 2010; Finsen and Owens, 2011) was the historic rationale for administering GCs as first line therapy following spinal cord injury (Bracken et al., 1984). However, Dex administration following traumatic injury to the zebrafish brain (Kyritsis et al., 2012) or spinal cord (Ohnmacht et al., 2016; Tsarouchas et al., 2018) is recognized to inhibit neural regeneration despite suppression of hematogenous cellular infiltration and microglial activation. The observed upregulation of pro-inflammatory cytokines, including *tumor necrosis factor* α (*tnfα*), was attributed to immune cell activation after zebrafish brain injury and recently in the spinal cord (Tsarouchas et al., 2018). The cellular sources of the pro-inflammatory signals were not determined in the brain (Kyritsis et al., 2012). In the injured spinal cord, *tnfα* expression was observed in macrophages, microglia, neutrophils, and other cell-types including neurons (Tsarouchas et al., 2018). However, the relevant GC target cells have not been examined in any previous study. Like other cytokines, Tnfα is pleiotropic and its production is not restricted to immune cells. For example, in the context of zebrafish retinal injury, Tnfα is required for triggering proliferation of radial Müller glia, and is expressed and secreted by neurons, not by microglia (Nelson et al., 2013). Furthermore, when combined with Notch suppression, administration of recombinant Tnfα is sufficient to stimulate Muller glial proliferation and neurogenesis (Conner et al., 2014). Our data from the “bloodless” zebrafish mutant concur with conclusions from published studies of zebrafish retinal injury, i.e., regeneration-initiating signals are not of microglial or hematogenous origin. Furthermore, the GC dose that we found inhibitory to functional regeneration in the present study was 50-fold lower than that used to suppress immune cell activity in larval spinal cord injury (Ohnmacht et al., 2016). Nevertheless, our data do not exclude an effect of GCs on other regenerative supporting processes.

Altogether, the data we present justify a revised model of GC signaling following spinal cord injury in which an inhibitory signaling cascade is triggered via GC receptors in radial ependymal glia. We have demonstrated that this signaling activity is constitutive in ependymal glia of zebrafish, and is down-regulated following cord injury. Independent of their immunosuppressive effects (Ohnmacht et al., 2016; Tsarouchas et al., 2018), GCs limit the pro-regenerative response of ependymal glia via direct stimulation of their receptors.

Despite lack of supportive evidence, and the recognized systemic complications of GC therapy (Coleman et al., 2000; Hurlbert, 2000; Short et al., 2000; Hurlbert and Hamilton, 2008), the practice of administering GCs in the clinical management of acute spinal cord injuries continues (Eck et al., 2006; Schroeder et al., 2014). Inhibitory mechanisms likely limit functional recovery in mammals, regardless of putative stimulatory factors. Our new data offer the challenge of designing preclinical studies in acute phase spinal injury to test the efficacy of targeted anti-GC therapy, with or without synergistic agents (e.g., recombinant fibroblast growth factor, Rabchevsky et al., 2000; Tsai et al., 2008; Goldshmit et al., 2014), to stimulate ependymal glia. The functional benefit of GC receptor suppression merits investigation.

## Ethics Statement

This study was carried out in accordance with the Mayo Clinic Institutional Animal Care and Use Committee. The protocol was approved by the Mayo Clinic Institutional Animal Care and Use Committee (IACUC protocol A36315-15; IACUC protocol A00001766-16).

## Author Contributions

CN and JH conceptualized the study and wrote the original draft of the manuscript. CN, NM, and JH contributed to the methodology. CN, HL, RK, and AK were responsible for the validation of data and investigated the study. CN contributed to the formal analysis. CN, HL, RK, NM, KC, VL, AK, and JH were responsible for editing and reviewing the manuscript. CN and AK visualized the study. KC, VL, AW, and JH supervised the study. JH contributed to the project administration. AW and JH were responsible for the funding acquisition.

## Conflict of Interest Statement

The authors declare that the research was conducted in the absence of any commercial or financial relationships that could be construed as a potential conflict of interest.

## Supplementary Material

FIGURE S1 NR3C1 Expression in the Spinal Cord of Neonatal Rats. **(A–C)** Neonatal (P0) rat spinal cord transverse sections examined at the T9 level. Immunostaining for NR3C1 (green), vimentin (red) and DAPI staining (blue). Base line Nr3c1 expression is present in ependymal glia **(A)**. Boxed region B is magnified 270% in **B^1,2^** and ependymal glial nuclei are marked with arrowheads. Boxed region C highlights NR3C1-positive non-ependymal glia (arrows) and is magnified 270% in **C^1,2^**. Scale bars, 50 μm **(A)** and 12.5 μm (**B^1^**, same as **B^2^–C^2^**). Representative images from six rat spinal cords (three sections per cord examined). ^∗^denotes central canal.

FIGURE S2 TALEN-mediated Downregulation of *nr3c1.* **(A,B)** Zebrafish spinal cord transverse sections. Embryos were injected with non-targeting GM2 **(A–A^2^)** or *nr3c1* targeting TALENs **(B–B^2^)**, then maintained for four dpf. Immunostaining for Nr3c1 (green) and Gfap (red), and DAPI staining (blue). Scale bar, 20 μm (**A** is the same as **B**). ^∗^denotes the central canal. Representative images from 10 spinal cords. The data support Figure 1, 2, 9 by confirming *nr3c1* mutation and specificity of Nr3c1 immunoreactivity.

FIGURE S3 Glucocorticoids do not Alter Cell Viability Following SCI. **(A–C)** Whole-mount preparations (lateral view) of wild-type larval zebrafish show TUNEL (red) and DAPI staining (blue) in the spinal cords from uninjured no treatment controls **(A)**, and 6 h post SCI with either no treatment **(B)** or +Dex **(C)**. Dashed boxes represent a 200-μm region centered at the lesion site used for quantifications and serve as scale bars (**A** is the same as **B,C**). **(D)** Quantification of the mean number of TUNEL-positive nuclei at 3-, 6-, 12-, 24-h post SCI and 6 h post SCI +Dex (± SEM). ^∗^*p* < 0.05, ^∗∗^*p* < 0.01 (compared to no injury); ns, not significant; 10 whole mounts per condition.
