# Supplementary figures and images for: Glucocorticoids Target Ependymal Glia and Inhibit Repair of the Injured Spinal Cord
Source: Front Cell Dev Biol. 2019 Apr 24;7:56. doi: 10.3389/fcell.2019.00056 (PMC6491705; doi:10.3389/fcell.2019.00056)

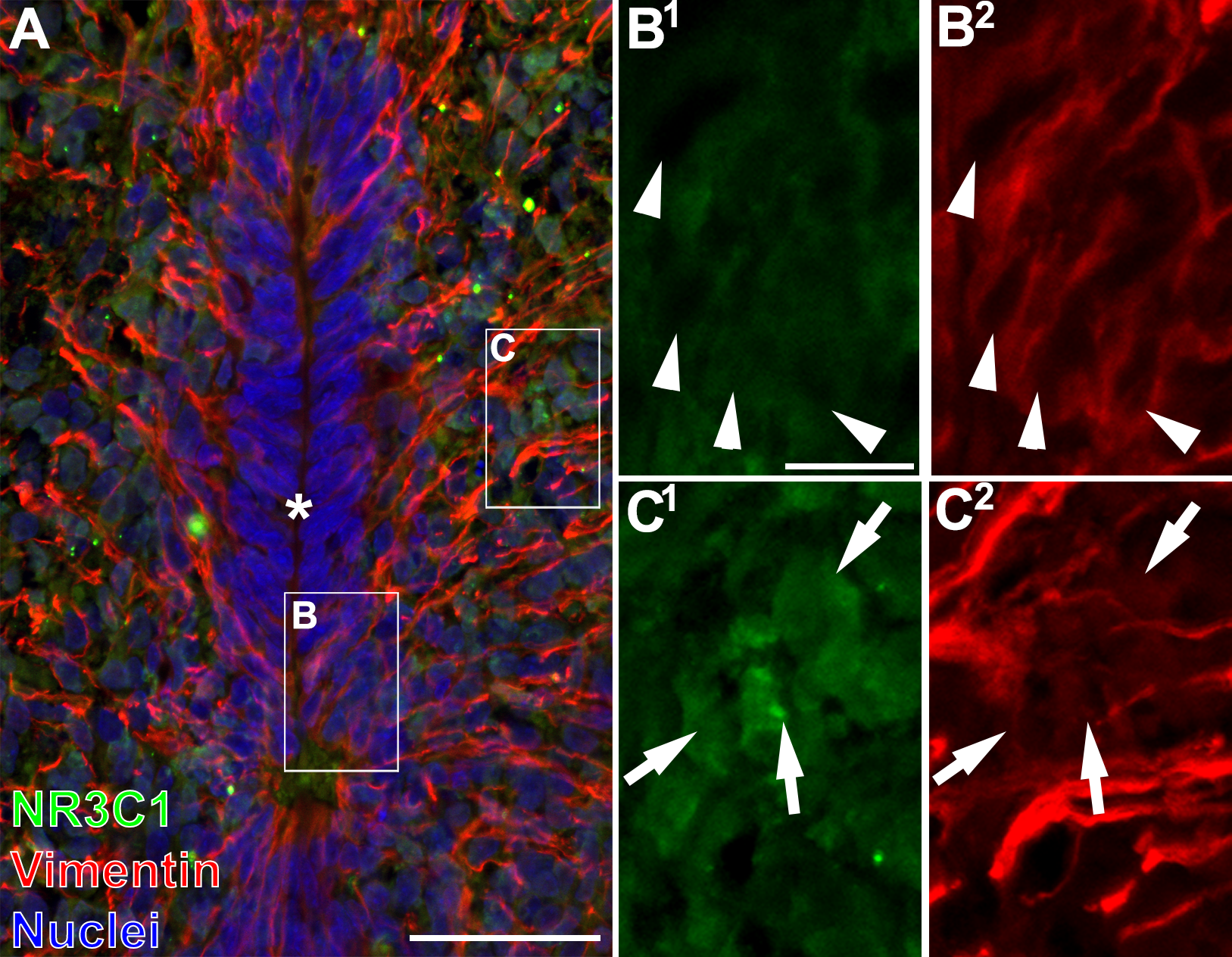

Supplement: FIGURE S1 — NR3C1 Expression in the Spinal Cord of Neonatal Rats. (A–C) Neonatal (P0) rat spinal cord transverse sections examined at the T9 level. Immunostaining for NR3C1 (green), vimentin (red) and DAPI staining (blue). Base line Nr3c1 expression is present in ependymal glia (A). Boxed region B is magnified 270% in B1,2 and ependymal glial nuclei are marked with arrowheads. Boxed region C highlights NR3C1-positive non-ependymal glia (arrows) and is magnified 270% in C1,2. Scale bars, 50 μm (A) and 12.5 μm (B1, same as B2–C2). Representative images from six rat spinal cords (three sections per cord examined). ∗denotes central canal. [file Image_1.TIF]

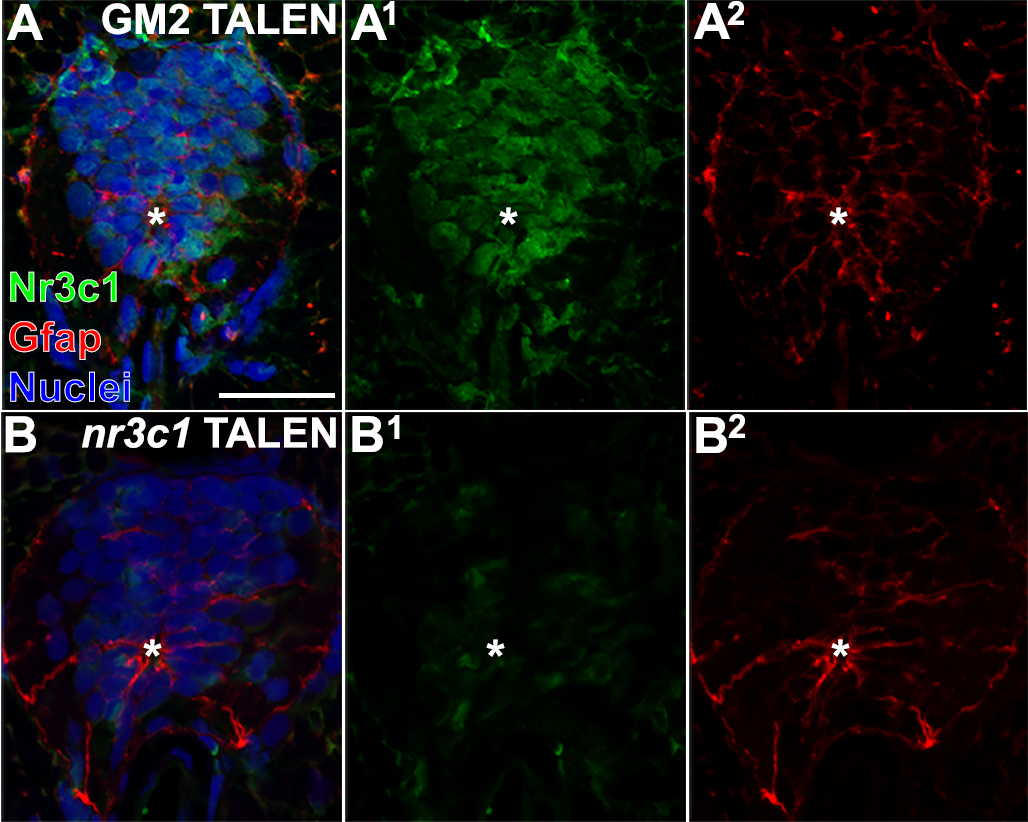

Supplement: FIGURE S2 — TALEN-mediated Downregulation of nr3c1. (A,B) Zebrafish spinal cord transverse sections. Embryos were injected with non-targeting GM2 (A–A2) or nr3c1 targeting TALENs (B–B2), then maintained for four dpf. Immunostaining for Nr3c1 (green) and Gfap (red), and DAPI staining (blue). Scale bar, 20 μm (A is the same as B). ∗denotes the central canal. Representative images from 10 spinal cords. The data support Figure 1, 2, 9 by confirming nr3c1 mutation and specificity of Nr3c1 immunoreactivity. [file Image_2.TIF]

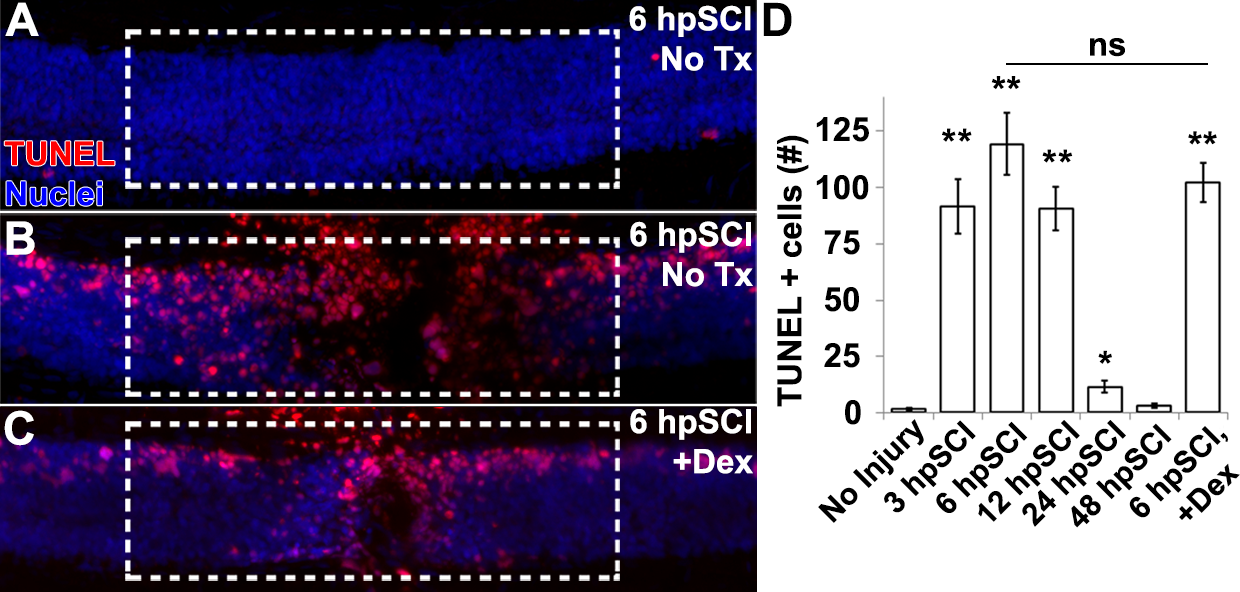

Supplement: FIGURE S3 — Glucocorticoids do not Alter Cell Viability Following SCI. (A–C) Whole-mount preparations (lateral view) of wild-type larval zebrafish show TUNEL (red) and DAPI staining (blue) in the spinal cords from uninjured no treatment controls (A), and 6 h post SCI with either no treatment (B) or +Dex (C). Dashed boxes represent a 200-μm region centered at the lesion site used for quantifications and serve as scale bars (A is the same as B,C). (D) Quantification of the mean number of TUNEL-positive nuclei at 3-, 6-, 12-, 24-h post SCI and 6 h post SCI +Dex (± SEM). ∗p < 0.05, ∗∗p < 0.01 (compared to no injury); ns, not significant; 10 whole mounts per condition. [file Image_3.TIF]
